# Minocycline prevents retinal inflammation and vascular permeability following ischemia-reperfusion injury

**DOI:** 10.1186/1742-2094-10-149

**Published:** 2013-12-10

**Authors:** Steven F Abcouwer, Cheng-mao Lin, Sumathi Shanmugam, Arivalagan Muthusamy, Alistair J Barber, David A Antonetti

**Affiliations:** 1 Department of Ophthalmology and Visual Sciences, University of Michigan Kellogg Eye Center, 1000 Wall Street, Ann Arbor, MI 48105, USA; 2 Department of Ophthalmology, Penn State University College of Medicine, 500 University Drive, Hershey, PA 17033, USA; 3 Department of Molecular and Integrative Physiology, University of Michigan, 1150 W. Medical Center Drive, Ann Arbor, MI 48109, USA

**Keywords:** Minocycline, Ischemia-reperfusion, Inflammation, Leukostasis, Blood-retinal barrier, Vascular permeability, Neurodegeneration

## Abstract

**Background:**

Many retinal diseases are associated with vascular dysfunction accompanied by neuroinflammation. We examined the ability of minocycline (Mino), a tetracycline derivative with anti-inflammatory and neuroprotective properties, to prevent vascular permeability and inflammation following retinal ischemia-reperfusion (IR) injury, a model of retinal neurodegeneration with breakdown of the blood-retinal barrier (BRB).

**Methods:**

Male Sprague–Dawley rats were subjected to 45 min of pressure-induced retinal ischemia, with the contralateral eye serving as control. Rats were treated with Mino prior to and following IR. At 48 h after reperfusion, retinal gene expression, cellular inflammation, Evan’s blue dye leakage, tight junction protein organization, caspase-3 activation, and DNA fragmentation were measured. Cellular inflammation was quantified by flow-cytometric evaluation of retinal tissue using the myeloid marker CD11b and leukocyte common antigen CD45 to differentiate and quantify CD11b^+^/CD45^low^ microglia, CD11b^+^/CD45^hi^ myeloid leukocytes and CD11b^neg^/CD45^hi^ lymphocytes. Major histocompatibility complex class II (MHCII) immunoreactivity was used to determine the inflammatory state of these cells.

**Results:**

Mino treatment significantly inhibited IR-induced retinal vascular permeability and disruption of tight junction organization. Retinal IR injury significantly altered mRNA expression for 21 of 25 inflammation- and gliosis-related genes examined. Of these, Mino treatment effectively attenuated IR-induced expression of lipocalin 2 (LCN2), serpin peptidase inhibitor clade A member 3 N (SERPINA3N), TNF receptor superfamily member 12A (TNFRSF12A), monocyte chemoattractant-1 (MCP-1, CCL2) and intercellular adhesion molecule-1 (ICAM-1). A marked increase in leukostasis of both myeloid leukocytes and lymphocytes was observed following IR. Mino treatment significantly reduced retinal leukocyte numbers following IR and was particularly effective in decreasing the appearance of MHCII^+^ inflammatory leukocytes. Surprisingly, Mino did not significantly inhibit retinal cell death in this model.

**Conclusions:**

IR induces a retinal neuroinflammation within hours of reperfusion characterized by inflammatory gene expression, leukocyte adhesion and invasion, and vascular permeability. Despite Mino significantly inhibiting these responses, it failed to block neurodegeneration.

## Introduction

The intraocular pressure-induced retinal ischemia-reperfusion (IR) model is a useful tool for studying the neuronal response to a transient ischemic injury. The model employs an ischemic period, typically ranging from 45 min up to 120 min, followed by natural reperfusion that leads to neurodegeneration (for review see [1]). Electroretinogram (ERG) analysis revealed significant decreases in neuronal function one week after IR, with reduced a-wave and b-wave amplitudes [2]. IR causes loss of neuronal cells indicated by decreased thicknesses of retinal layers, including the ganglion cell layer (GCL), inner nuclear layer (INL) and inner plexiform layer (IPL) [3,4]. The apoptotic death of neurons in these layers is indicated by terminal deoxynucleotidyl transferase-mediated dUTP nick-end labeling (TUNEL) [5].

Recently it has been recognized that the IR model also recapitulates changes in the blood-retinal barrier (BRB) and retinal capillary degeneration observed in diabetic retinopathy and vein occlusions. Using optical coherence tomography (OCT), Kim and co-workers [6] recently demonstrated retinal thickening indicative of edema in mice 3 d following IR, which was followed by continuous retinal layer thinning for as long as 4 wk after IR. In addition, IR injury to rats caused a rapid breakdown of the BRB, with markedly increased retinal vascular permeability 4 to 48 h following ischemia [7,8]. Finally, retinal IR injury to rats induced a loss of vascular cells occurring 7 to 14 days following reperfusion [9-11].

Much less is known about the inflammatory response to retinal IR injury. Several studies have documented an induction of pro-inflammatory genes in rodent retinas following IR, including intracellular adhesion molecule ICAM-1 and chemoattractants such as CCL2 [11-17]. However, there are few studies examining the consequences of inflammatory gene expression in IR injury. The accumulation of leukocytes in retinal tissue after IR has been quantified by nonspecific staining methods [18-20] and qualitatively observed by immunohistochemistry with antibodies to leukocyte antigens [21,22], but the characteristics of this leukostasis have not been examined. Of particular interest is how this inflammatory response relates to neuronal and vascular injury.

Minocycline is a blood–brain barrier (BBB) permeable tetracycline derivative that exhibits anti-inflammatory, anti-apoptotic and antioxidant properties, and which inhibits neuroinflammation and neurodegeneration in the central nervous system (CNS) (for recent reviews see [23,24]). Mino inhibits retinal neurodegeneration in several models of retinopathies, including light-induced injury, axotomy, experimental glaucoma, photoreceptor degeneration, diabetic retinopathy, and IR injury [25-32]. In the present study we evaluated the abilities of Mino to affect vascular permeability, inflammation and neurodegeneration in a rat model of retinal IR employing a relatively short, 45 min, period of elevated intraocular pressure (IOP). Mino treatment inhibited the induction of a subset of IR-responsive genes associated with neuroinflammation, diminished the retinal accumulation of inflammatory myeloid leukocytes and lymphocytes, and reduced retinal vascular permeability following IR. In contrast, Mino did not significantly affect cell death following IR, suggesting that the anti-inflammatory and anti-permeability effects of Mino were disassociated from neuroprotection.

## Methods

### Retinal ischemia-reperfusion and minocycline treatments

Male Sprague–Dawley rats (Charles River Laboratories, http://www.criver.com) were maintained under specific pathogen-free conditions and monitored by quarterly sentinel testing and treated in accordance with the guidelines of the University of Michigan School of Medicine and Penn State Hershey College of Medicine Institutional Animal Care and Use Committees. Ischemia was applied to the left eyes of rats weighing between 200 g and 225 g by increasing the intraocular pressure to cut off the retinal arterial blood supply as previously described [7]. Elevated pressures were maintained for 45 min before removing needles and allowing natural reperfusion. Sham eyes were treated by briefly inserting a 32-gauge needle into the anterior chamber of the eye through the cornea. Unless otherwise stated, animals were euthanized and retinas removed for analysis at 48 h following IR. Mino (Sigma-Aldrich Chemical Co., http://www.sigmaaldrich.com) was dissolved fresh in phosphate-buffered saline (PBS) and adjusted to neutral pH immediately prior to administration. We employed a Mino treatment regimen used in several previous studies (see [24]). Mino was delivered as twice-daily intraperitoneal (IP) injections, with two initial dosages of 45 mg/kg one day prior to ischemia and dosages of 22.5 mg/kg just prior to ischemia and every 12 h for the next 2 d during the reperfusion period. Final injections were given 1 h prior to harvesting retinas for neurodegeneration or inflammation assays, or 1 h prior to injection of Evan’s blue dye for permeability assays. No treatment controls received equal volume injections of PBS. For a dose–response experiment Mino was similarly delivered twice daily, but at consistent dosages of 22.5, 7.5 or 2.5 mg/kg. (For intravitreal injection of Mino methods used in Supplemental Data see Additional file 1: Supplemental Methods).

### Retinal permeability assessment

Accumulative blood-retinal barrier leakages were measured using the Evans blue dye method of Xu and co-workers [33] as previously described [7]. The dye binds tightly to serum albumin, thus indicating the leakage of albumin across the blood-retinal barrier. Statistical differences between like-treated Sham and IR retina groups were analyzed by paired Student’s *t*-test, with effects of treatments analyzed by unpaired Student’s *t*-test.

### Retinal whole mount immunofluorescence

Eyes were removed, punctured and fixed in 4% paraformaldehyde in PBS for 15 min at room temperature (RT) before dissecting retinas. Retinas were removed by orbital dissection, rinsed in tris-buffered saline containing 0.3% Triton X100 (TBST) and blocked by incubation in TBST containing 10% goat serum for 1 h at RT. To examine leukostasis and leukocyte invasion, retinas were incubated with mouse anti-rat CD45 (1:50, BD Biosciences, http://www.bdbiosciences.com) in TBST plus goat serum for 3 days at 4°C followed by extensive rinsing in TBST for 24 h. Retinas were then incubated with Alexa Fluor-647-labeled goat anti-mouse IgG (1:1000, Invitrogen-Life Technologies, lifetechnologies.com), Alexa Flour488-labeled isolectin B4 (IB4) from *Griffonia simplicifolia* (1:75, Invitrogen-Life Technologies, lifetechnologies.com) and 10 μg/ml Hoechst-33342 DNA stain (Invitrogen-Life Technologies, lifetechnologies.com) in TBST for 24 h at RT followed by extensive rinsing in TBST for 24 h. To examine endothelial tight junction organization, retinas were incubated with rabbit anti-Zonula occludens 1 (ZO-1) antibody (1:50, Invitrogen-Life Technologies, lifetechnologies.com) and then with Alexa Fluor 594-conjugated anti-rabbit IgG secondary antibody (1:1000, Invitrogen-Life Technologies, lifetechnologies.com). Retinas were flat mounted on 3-aminopropyltriethoxysaline-coated slides with Prolong Gold mounting media (Invitrogen-Life Technologies, lifetechnologies.com). Images were acquired with a Leica TCS SP5 AOBS confocal microscope (http://www.leica-microsystems.com).

### Vascular endothelial cell border organization grading

Confocal *Z*-stacks of 10 images collected over a depth of 5 μm were projected as one composite image. Loss of vascular ZO-1 organization at endothelial cell borders was quantified by a semi-quantitative rank scoring system based on a scale of 1 to 5, with 1 corresponding to complete loss of continuous border staining and 5 corresponding to entirely continuous border staining. Scoring was completed in a masked fashion by three impartial observers who were provided scored images for reference. For each group or retinas, the scores for 8 to 18 images from three independent experiments were obtained and the frequencies for each ranking score calculated and plotted. Statistical differences between mean values of rank scores for each group were analyzed by one-way ANOVA.

### Examination of retinal gene expression by quantitative real-time polymerase chain reactions

Retinas were removed, flash-frozen in liquid nitrogen, and stored at -80°C until analysis. Total RNA was purified from retinal tissues using RNeasy Plus™ Mini kit (Qiagen, http://www.qiagen.com) with and QiaShredders™ (Qiagen, http://www.qiagen.com). Quantitative real-time polymerase chain reaction (qRT-PCR) was performed by reverse transcription of 0.8-1.0 μg of total RNA using random hexamers and oligo-dT primers in the presence of RNase inhibitor (Omniscript™ RT kit, Qiagen, http://www.qiagen.com). Duplex qPCRs were performed using the equivalent of 1 μl of RT reaction with gene-specific primers and FAM-labeled probes (Applied Biosystems Life Technologies, lifetechnologies.com), along with β-actin-specific primers and VIC-labeled probes (primer limited formulation, Applied Biosystems Life Technologies, lifetechnologies.com) and TaqMan™ Universal PCR master mix (Applied Biosystems Life Technologies, lifetechnologies.com). Primer-probe assay information and measured efficiencies obtained in duplex reactions are provided in (Additional file 2: Table S1). Reactions were performed and monitored using a CFX384 real time PCR system (BioRad, http://www.bio-rad.com). Relative normalized mRNA levels were calculated using the ΔΔC_t_ method. Statistical differences between like-treated Sham and IR retina groups were analyzed by paired Student’s *t*-test, with effects of treatments analyzed by unpaired Student’s *t*-test.

### Analysis of leukostasis and leukocyte infiltration by flow cytometry

Retinas were removed by orbital dissection and IR and sham retinas pooled (2 to 4 retinas per group). Tissues were diced with a scalpel into <1 mm pieces and then centrifuged at 400 ×g for 5 minutes at RT. Pelleted tissues were resuspended in a total of 500 μL of Hepes-buffered saline (HBSS) with 0.5 mg/ml of Liberase enzyme mix (TM research grade, Roche Applied Science, http://www.roche-applied-science.com) and 0.1 mg/ml DNase (Bovine Grade II, Roche Applied Science, http://www.roche-applied-science.com) and incubated at 37°C for 25 to 30 min with occasional agitation. After incubation, 10 ml of Dulbecco’s modified Eagles media (DMEM) containing 10% fetal bovine serum (FBS) was added to the reaction and retinal cells were forcibly strained three times through a 40-μM nylon mesh strainer using the rubber end of a syringe plunger. The strained cell suspension was centrifuged at 400 ×g for 5 min at RT and the pellet resuspended in 5 ml of HBSS. To remove clumps of cells, the suspension was centrifuged at 50 ×g for 1 min at RT, and the pellet discarded. The supernatant was centrifuged at 400 ×g for 5 min at RT and the cellular pellet resuspended in 500 μl of HBSS and transferred to several wells of a U-bottomed 96-well plate for antibody staining. Cells were centrifuged, pellets resuspended in 50 μl of PBS containing 20% rat serum and 1 μg/ml Fc-blocking antibody (mouse anti-rat FcγII/CD32, clone D34-485, BD Biosciences, http://www.bdbiosciences.com) and incubated for 20 min on ice. Biotin-conjugated anti-rat RT1B/MHCII antibody (1:10 volume, clone OX-6, Serotec, http://www.abdserotec.com) was added and the incubation was continued for 45 min on ice. After rinsing three times in PBS, cells were resuspended in PBS containing 20% rat serum with RPE-conjugated anti-rat CD11b monoclonal antibody (1:50 volume, clone OX-42, AbD Serotec, http://www.abdserotec.com), FITC-conjugated anti-rat CD45 monoclonal antibody (1:50 volume, clone OX-1, AbD Serotec, http://www.abdserotec.com), and APC-Cy7-conjugated streptavidin (1:100 volume, BD Biosciences, http://www.bdbiosciences.com) and incubated for 45 min on ice. After being rinsed, cells were analyzed using an LSRII flow cytometer (BD Biosciences, http://www.bdbiosciences.com). CompBeads (BD Biosciences, http://www.bdbiosciences.com) anti-mouse Ig particle compensation set was incubated with each antibody or biotin-avidin-antibody pair for compensation corrections for spectral overlaps. Cytometer data was analyzed using FlowJo software (Tree Star Inc., http://www.treestar.com). To avoid events representing debris and clumps of cells, events were gated from scatter plots of forward and side scatter in identical fashions for each group prior to analysis of CD11b, CD45 and MHCII immunostaining intensities. Statistical differences between like-treated Sham and IR retina groups were analyzed by paired Student’s *t*-test, with effects of treatments analyzed by unpaired Student’s *t*-test.

### Evaluation of retinal cell death

Caspase-3 (DEVDase) activity was measured in retinal homogenates using the fluorometric CaspACE assay system (Promega, http://www.promega.com) as previously described [7]. Apoptotic DNA cleavage was assayed using a Cell Death Detection ELISA Kit (Roche Applied Science, http://www.roche-applied-science.com) and normalized to retinal wet weight as previously described [7]. Statistical differences between like-treated Sham and IR retina groups were analyzed by paired Student’s *t*-test, with effects of treatments analyzed by unpaired Student’s *t*-test. (For additional retinal cell methods used in Supplemental Data see Additional file 1: Supplemental Methods).

## Results

### Minocycline treatment inhibited retinal vascular permeability following ischemia-reperfusion

**Figure 1:**
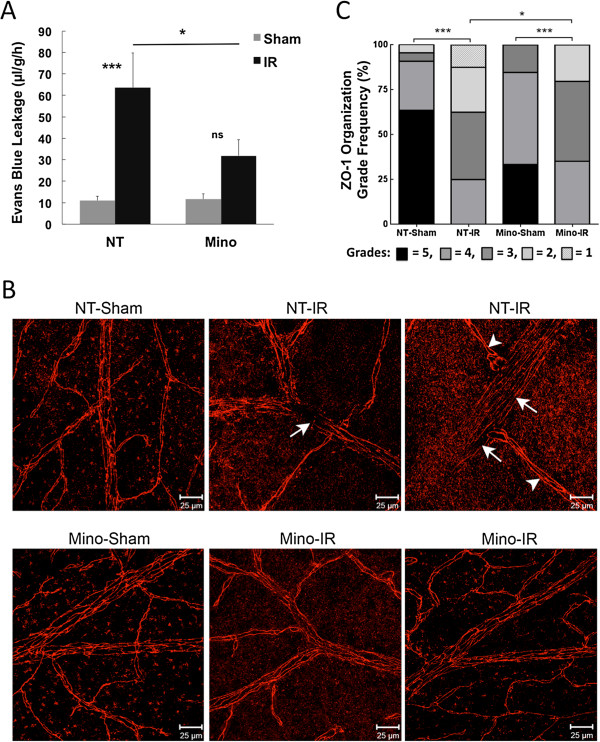
**Minocycline (Mino) treatment significantly inhibited retinal vascular leakage and tight junction reorganization following ischemia-reperfusion (IR).** Mino was delivered as twice-daily intraperitoneal (IP) injections, with two initial dosages of 45 mg/kg prior to ischemia and dosages of 22.5 mg/kg just prior to ischemia and every 12 h for the next 2 d during the reperfusion period as described in Materials and Methods. Non-treated (NT) animals received PBS vehicle injections. One eye of each rat was subjected to IR for 45 min or needle puncture only (Sham) and after 48 h of reperfusion retinas were assayed for **(A)** Evans blue dye leakage (n = 8 retinas per group), or **(B and C)** for vascular endothelium tight junction organization by immunohistochemistry of ZO-1 protein at endothelial cell borders (n = 4 retinas per group). Representative images from Sham and IR retinas are shown in **B**, with arrowheads indicating intact borders and arrows indicating regions of ZO-1 discontinuity. Distributions of endothelial cell border organization grading frequency are shown in **C**, with five representing fully continuous border staining and one representing complete loss of continuous border staining. *P ≤0.05, **P ≤0.01 and *** *P* ≤0.001 by Student’s *t*-test.

Using a rat model of IR injury caused by 45 min of ischemia, we previously demonstrated that both retinal neurodegeneration and increased vascular permeability occurs at 4 h to 48 h following IR [7]. We hypothesized that Mino could protect against vascular dysfunction in this model, and, thus, effects of Mino treatment on the retinal vascular leakage after 48 h of reperfusion were tested. We chose to use a treatment regimen employing twice-daily IP injections of Mino with two initial loading doses of 45 mg/kg followed by doses of 22.5 mg/kg, which has been used in several previous rat studies of ischemic injury and neurodegeneration (listed in [24]). Mino treatment significantly (*P* <0.05) inhibited the increase in retinal Evans blue dye accumulation, a measure of vascular albumin leakage, at 48 h after IR by 61% (Figure 1A). In addition, we found that intravitreal injection of Mino (640 ng/eye injected 1 h before and 4 h after IR) also significantly (*P* <0.05) inhibited the vascular permeability increase 24 h following IR to a very similar extent (77%) as observed with systemic Mino treatment (see Additional file 3: Figure S1A). These data suggest that Mino acts locally to reduce retinal permeability at 24 to 48 h after IR. However, when the effect of Mino treatment on vascular permeability was examined immediately following IR, the drug had no significant effect (see Additional file 4: Figure S2).

ZO-1 represents a central organizing protein in the junction complex comprising the BRB [34]. To assess organization of the endothelial tight junction complex, localization of ZO-1 was imaged in retinal flat mounts by immunofluorescence (IF) and confocal microscopy. At 48 h following IR ZO-1 localization was apparently altered specifically at arterioles in this IR model (Figure 1B). Further, Mino treatment significantly reversed the perturbation of ZO-1 localization, as indicated by masked image scoring performed by impartial evaluators (Figure 1C).

### Minocycline treatment inhibited expression of ischemia-reperfusion-responsive genes associated with neuroinflammation, but not those associated with astrogliosis following ischemia-reperfusion

To examine the effect of Mino treatment on the inflammatory response following IR we used qRT-PCR analysis to examine the expression levels of 25 mRNAs at 48 h following IR in rats systemically treated with or without Mino as described above. The mRNAs examined were chosen from a previously obtained transcriptomics data set for this rat IR model [7], with preference for genes associated with inflammation and astrogliosis (see Additional file 2: Table S1). For comparison, the induction of expression of each of these mRNAs in retinas 4 h following intravitreal injection of 1 μg/eye of lipopolysaccharide (LPS) was also examined. Retinal expression of 21 of these mRNAs were significantly altered by IR, with 20 mRNAs increased from 89% (CHI3L1) to 47.6 fold (CCL2) and only glutamate ammonia ligase (GLUL) significantly decreased 55% by IR (see Additional file 2: Table S1). Significantly increased mRNA expression was observed for several pro-inflammatory genes, including: C-X-C motif chemokine ligand 2 (CXCL2, 5.3-fold, *P* <0.001), interleukin-6 (IL6, 9.5-fold, *P* <0.001, interleukin-1beta (IL1B, 7.5-fold, *P* = 0.03), tumor necrosis factor alpha (TNF, 9.1-fold, *P* <0.001), C-X-C motif chemokine ligand 1 (CXCL1, 5.5-fold, *P* <0.001), C-C motif chemokine ligand 3 (CCL3, 9.6-fold, *P* <0.001) and CD14 molecule (22.2-fold, *P* <0.001). In contrast, expression of three mRNAs that are characteristic of classical inflammation, C-X-C motif chemokine ligand 10 (CXCL10), inducible nitric oxide synthase 2 (NOS2, iNOS) and prostaglandin-endoperoxide synthase 2/cyclooxygenase 2 (PTGS2, COX2), were not significantly altered at 48 h after IR. However, the retinal levels of these three mRNAs were significantly increased by LPS injection by 4700-fold (*P* = 0.05), 63-fold (*P* <0.001) and 9.4-fold (*P* <0.001), respectively. Expression of mRNAs representing two markers of astrogliosis, glial fibrillary acidic protein (GFAP) and vimentin (VIM), were significantly (*P* <0.001) increased by 6.8-fold and 3.0-fold, respectively, by IR. Neither of these mRNAs was responsive to LPS injection.

**Figure 2:**
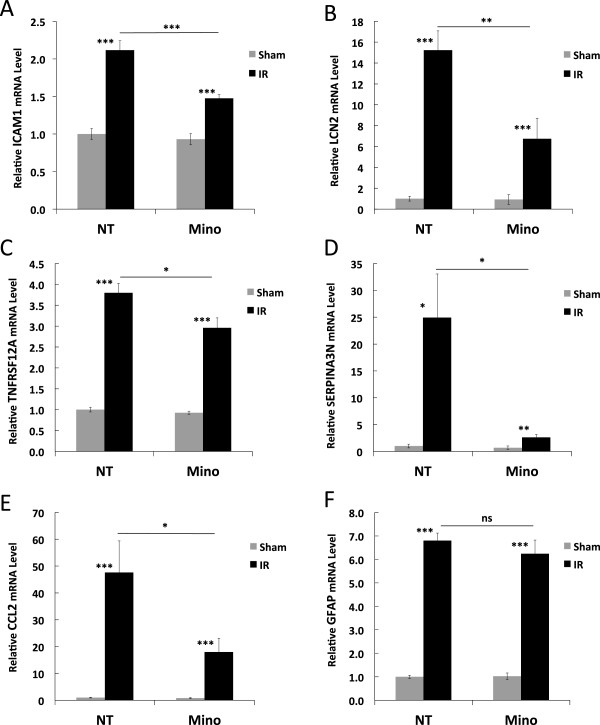
**Minocycline (Mino) treatment significantly diminished neuroinflammatory gene expression following ischemia-reperfusion (IR) without affecting astrogliosis-related gene expression.** Mino was delivered as twice-daily intraperitoneal (IP) injections, with two initial dosages of 45 mg/kg prior to ischemia and dosages of 22.5 mg/kg just prior to ischemia and every 12 h for the next 2 d during the reperfusion period as described in Materials and Methods. Non-treated (NT) animals received PBS vehicle injections. One eye of each rat was subjected to IR for 45 min or needle puncture only (Sham) and after 48 h of reperfusion total RNA was isolated from retinas and relative mRNA levels of neuroinflammation-related genes **(A)** ICAM-1, **(B)** LCN2, **(C)** TNFRSR12A, **(D)** SERPINA3N, **(E)** CCL2 and **(F)** the astrogliosis-related gene GFAP by duplex qRT-PCR with β-actin mRNA serving as control. Results shown are the means and standard error of means obtained from eight animals per group. *P ≤0.05, **P ≤0.01 and ***P ≤0.001 by Students *t*-test.

Comparing mRNA levels in sham-treated eyes from rats treated with and without Mino revealed that drug treatment alone significantly affected the expression of five mRNAs, including IL6 (increased 2.5-fold, *P* = 0.01), C-C motif chemokine ligand 5 (CCL5, decreased 35%, *P* = 0.05), endothelin 2 (EDN2, decreased 49%, *P* = 0.05), CCL3 (decreased 30%, *P* = 0.03) and the lectin galectin binding soluble 3 (LGALS3, decreased 70%, *P* = 0.002) see (Additional file 2: Table S1). Mino also significantly inhibited the IR-induced expression of 5 genes (Figure 2). These included ICAM-1 (51% inhibition, *P* <0.001), lipocalin 2 (LCN2, 59% inhibition, *P* = 0.008), serpin peptidase inhibitor clade A member 3 N (SERPINA3N, 91% inhibition, *P* = 0.02), TNF receptor superfamily member 12A (TNFRSF12A, 27% inhibition, p = 0.03) and CCL2 (63% inhibition, *P* = 0.04). The inhibitory effects of Mino treatment on the IR responses of several other pro-inflammatory genes were nearly significant. These included: CXCL2 (54% inhibition, *P* = 0.06), IL6 (63% inhibition, *P* = 0.06), IL1B (82% inhibition, *P* = 0.07), and TNF (30% inhibition, *P* = 0.07) (see Additional file 2: Table S1). In contrast, Mino treatment did not significantly affect the IR responses of GFAP (Figure 2F) and VIM (see Additional file 2: Table S1), with calculated inhibitions of 10% and 2%, respectively. Thus, Mino had a generally inhibitory effect on the expression of pro-inflammatory genes following IR without altering the astrogliosis response to retinal IR injury.

### Minocycline treatment inhibited cellular inflammation following ischemia-reperfusion

**Figure 3:**
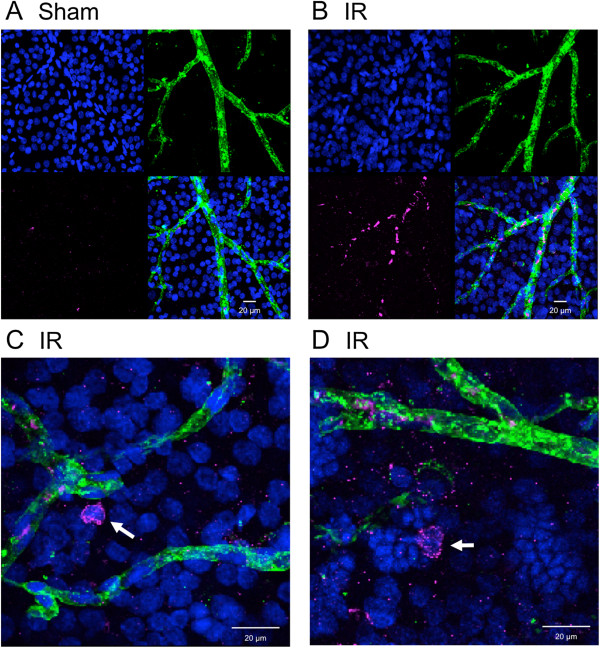
**Leukostasis and retinal tissue invasion of CD45-positive leukocytes following ischemia-reperfusion (IR).** Eyes were subjected to IR for 45 min or needle puncture only (Sham) and after 48 h of reperfusion retinas were removed, stained with antibodies to CD45 (magenta), isolectin B4 (IB4, green) and Hoechst nuclear stain (blue) and then flat mounted. Representative images showing CD45-staining leukocytes within IB4-stained vessels from IR retina **(A)** and sham retina **(B)** are shown along with magnified images **(C and D)** of CD45-positive cells (arrows) found within retinal tissue from IR retinas.

Given that Mino reduced the overall transcriptional pro-inflammatory response to IR and significantly inhibited the induction of expression of the monocyte chemoattractant CCL2 as well as the leukocyte adhesion molecule ICAM-1, we hypothesized that Mino treatment inhibits the attraction, vascular adherence and invasion of monocytes to retinal tissue following IR, thus leading to a diminished cellular inflammation. Figure 3 shows representative images of retinal flat mounted Sham and IR retinas harvested 48 h after IR and probed with antibody to CD45 to detect leukocytes and with IB4 lectin, which binds with high affinity to endothelial cells [35] and also labels infiltrating neutrophils [36]. The appearance of CD45-positive leukocytes was apparent in IR retinas, with the vast majority of these cells found within the vascular lumen and most abundant within intermediate-sized arterioles and venules. These cells were essentially absent in Sham retinas. A few amoeboid cells exhibiting uniform peripheral CD45 staining were also identified within IR injured retinas; these were not found in retinas from Sham treated eyes. Although microglia express low levels of CD45, no cells with ramified morphology resembling microglia were observed to exhibit CD45 staining above background levels in either Sham or IR retinas. Thus, this qualitative analysis demonstrated an obvious leukostasis with occasional tissue invasion of leukocytes 48 h after IR.

**Figure 4:**
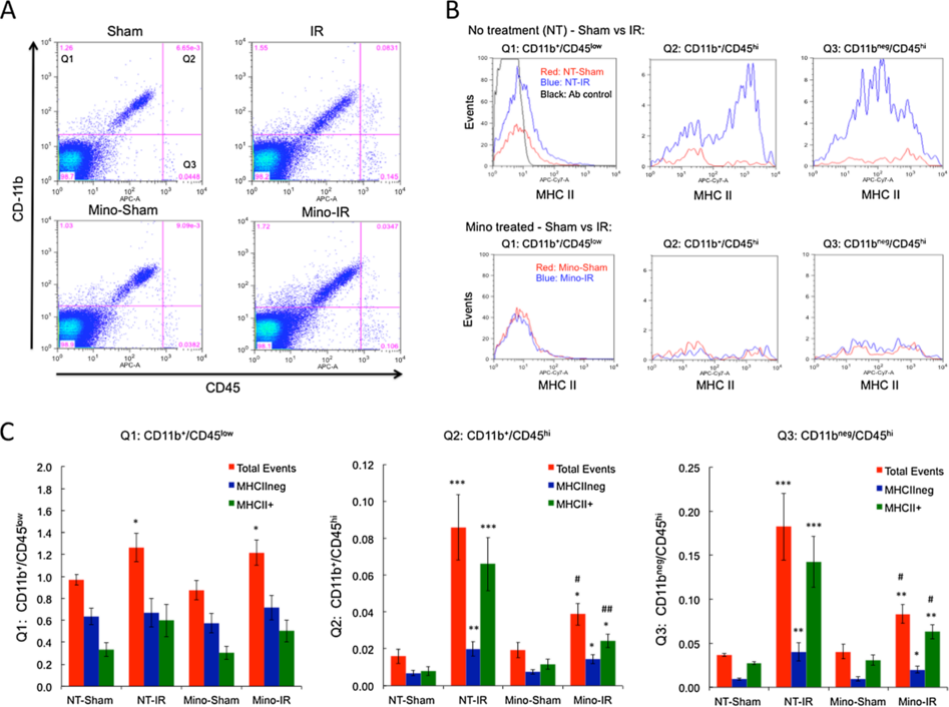
**Minocycline (Mino) treatment significantly diminishes cellular inflammation following ischemia-reperfusion (IR).** Mino was delivered as twice-daily intraperitoneal (IP) injections, with two initial dosages of 45 mg/kg prior to ischemia and dosages of 22.5 mg/kg just prior to ischemia and every 12 h for the next 2 d during the reperfusion period. Non-treated (NT) animals received PBS vehicle injections. One eye of each rat was subjected to IR for 45 min or needle puncture only (Sham) and after 48 h of reperfusion retinas were enzymatically dissociated and cells analyzed by flow cytometry. **(A)** Representative scatter plots of CD11b and CD45 immunostaining intensities of total retinal cells from Sham and IR retinas of rats treated with PBS or Mino. Events are partitioned into quadrant 1 (Q1) containing CD11b^+^/CD45^low^ cells, quadrant 2 (Q2) containing CD11b^+^/CD45^hi^ cells and quadrant 3 (Q3) containing CD11b^neg^/CD45^hi^ cells. The fourth quadrant contains the majority of retinal cells, which are CD11b^neg^/CD45^neg^. **(B)** Example of MHCII staining distributions of cells from quadrants 1, 2 and 3. Red traces are for cells from Sham-treated retinas, blue traces are for cells from IR retinas and the black trace is for cells incubated without the MHCII antibody and used to define the intensity cutoff between define MHCII^+^ and MHCII^neg^ cells. **(C)** Quantification of total events, MHCII^+^ events and MHCII^neg^ events from quadrants 1, 2 and 3 from Sham and IR retinas from PBS- and Mino-treated rats. The numbers shown are mean percentiles relative to all events, including those in quadrant 4, obtained from three separate flow experiments, with a total of 11 to 12 determinations per group. Significant differences between Sham and IR groups are indicated as *P ≤0.05, **P ≤0.01 and ***P ≤0.001, while significant effects of Mino-treatment are indicated by #*P* ≤0.05 and ##*P* ≤0.01. All comparisons are by Students *t*-test.

Next we used flow cytometry to quantify and better characterize this leukostasis response, as well as to test the effects of Mino treatment on this cellular inflammation at 48 h following IR (Figure 4). By probing all retinal cells for surface expression of CD45 and the myeloid marker CD11b the CD11b^+^/CD45^low^ cells (evidently microglia), CD11b^+^/CD45^hi^ cells (myeloid leukocytes) and CD11b^neg^/CD45^hi^ cells (non-myeloid leukocytes, aka lymphocytes) were gated and each population quantified relative to the total number of retinal cells (Figure 4A). To obtain information regarding the inflammatory state of the cells, the populations were also gated by surface expression of MHCII with MHCII^neg^ and MHCII^+^ subpopulations quantified (Figure 4B). Figure 4C indicates that IR caused a slight (30%) but significant (*P* <0.05) increase in the average total fraction of CD11b^+^/CD45^low^ cells (MHCII^neg^ plus MHCII^+^). CD11b^+^/CD45^low^ cells exhibited a unimodal distribution of MHCII content with approximately 2/3 of cells exhibiting an MHCII-negative phenotype and 1/3 with MHCII antibody staining intensity only slightly above that of the no antibody control. This distribution was not obviously altered by IR and the mean fluorescence intensity of MHCII antibody staining was not significantly altered (data not shown). However, the slight increase in CD11b^+^/CD45^low^ cell numbers following IR was primarily due to an increase of cells exhibiting low MHCII immunoreactivity. Mino treatment had no significant effects on the retinal microglial numbers in sham treated or IR injured retinas.

Consistent with the IF results, the relative numbers of all CD45-positive leukocytes were markedly increased by IR. This was due to significant increases (*P* <0.001) of both CD11b^+^/CD45^hi^ myeloid cells (increased more than 5-fold from 0.016 to 0.086% of all events) and CD11b^neg^/CD45^hi^ lymphocytes (increased nearly 5-fold from 0.037% to 0.18%) following IR (Figure 4C). Thus, the CD45-positive leukocytes accumulating in the retina following IR were composed of approximately 1/3 myeloid cells and 2/3 lymphocytes. CD11b^+^/CD45^hi^ myeloid cells exhibited a clear bimodal distribution of MHCII-content, which was particularly evident following IR due to a large increase in cells with a relatively high MHCII expression. IR significantly (*P* <0.001) increased MHCII^+^ myeloid leukocytes by more than 8-fold (from 0.008 to 0.066%). IR also significantly (*P* <0.01) increased the number of MHCII^neg^ myeloid leukocytes, but only by 3-fold (from 0.007 to 0.020%). In contrast to myeloid cells, CD11b^neg^/CD45^hi^ lymphocytes in the retina exhibited a broad unimodal MHCII distribution. Although separation of this population by MHCII expression was thus less practical, gating into MHCII^+^ and MHCII^neg^ populations was done for sake of comparison. IR significantly (*P* <0.001) increased accumulation of nominally MHCII^+^ myeloid leukocytes by 5-fold (from 0.028 to 0.14%), and significantly (*P* <0.01) increased MHCII^neg^ myeloid leukocytes by more than 4-fold (from 0.009 to 0.040%). Thus, the majority of myeloid and non-myeloid leukocytes accumulating in the retina after IR were MHCII-positive. Collectively, this data revealed that both myeloid and lymphocytic cells are significantly increased in the retina 48 h after IR with a predominant increase in MHCII^+^ cells, while IR had a limited effect on the number of resident microglia.

Mino treatment significantly inhibited the increase in both myeloid leukocytes and lymphocytes following IR (Figure 4). In Mino-treated rats the increase in CD11b^+^/CD45^hi^ myeloid cell numbers following IR was significant (*P* <0.05) but much lower than that observed in non-treated rats (2-fold versus 5-fold), corresponding to a 72% inhibition by Mino (*P* <0.05). The increase of CD11b^neg^/CD45^hi^ lymphocytes was also significant (*P* <0.01) in Mino-treated rats, but similarly reduced by 71% by Mino (2-fold versus nearly 5-fold). Gating of populations by MHCII expression revealed that the inhibitory effect of Mino treatment was biased toward MHCII^+^ leukocytes. Mino significantly (*P* <0.01) inhibited the increase of CD11b^+^/CD45^hi^/MHCII^+^ myeloid leukocytes by nearly 80% following IR. In contrast, Mino nominally inhibited the accumulation of MHCII^neg^ myeloid leukocyte population following IR by only 45% (*P* = 0.25). With Mino treatment the accumulation of CD11b^neg^/CD45^hi^/MHCII^+^ lymphocytes in response to IR was significantly reduced by 72% (*P* <0.05) compared to non-treated rats. For MHCII^neg^ lymphocytes, the calculated inhibition by Mino was similarly 70%, however, the effect of Mino did not reach significance (*P* = 0.08). In sum, Mino dramatically reduced the increase in myeloid cells and lymphocytes following IR with the prevalent effects being on MHCII^+^ populations.

### Minocycline did not significantly inhibit neurodegeneration following ischemia-reperfusion

**Figure 5:**
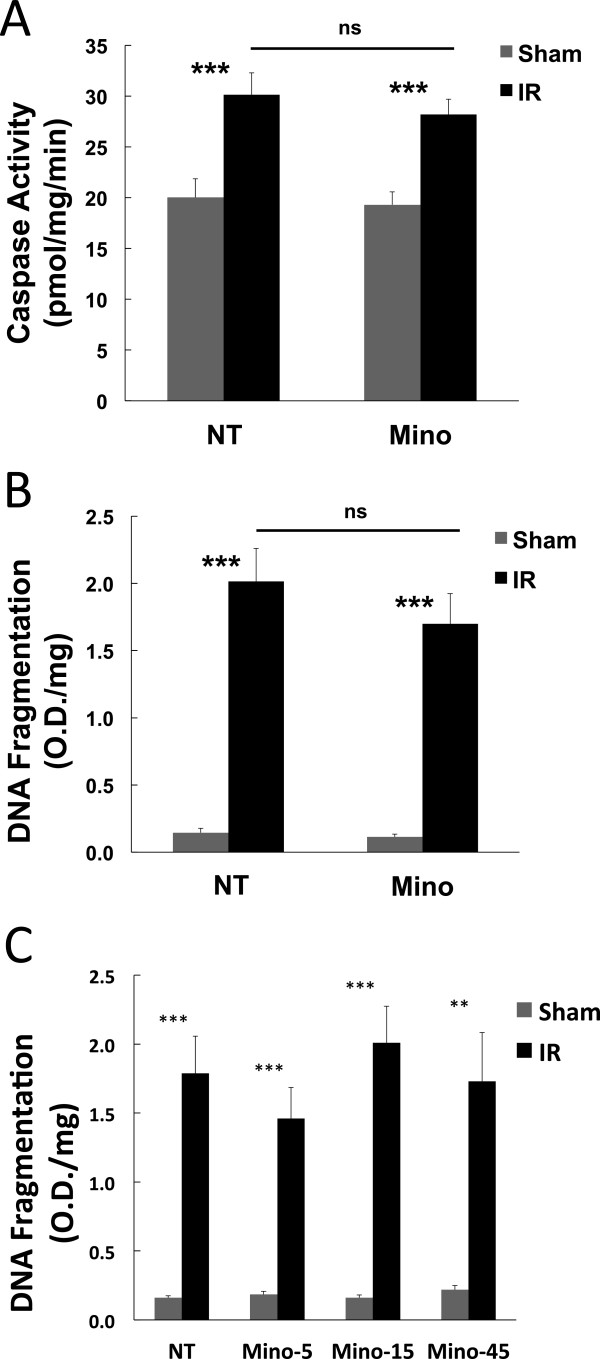
**Minocycline (Mino) treatment did not prevent cell death following ischemia-reperfusion (IR). (A and B)** Mino was delivered as twice-daily intraperitoneal (IP) injections, with two initial dosages of 45 mg/kg prior to ischemia and dosages of 22.5 mg/kg just prior to ischemia and every 12 h for the next 2 d during the reperfusion period as described in Materials and Methods. One eye of each rat was subjected to IR for 45 min or needle puncture only (Sham) and after 48 h of reperfusion retinas were assayed for **(A)** caspase-3 activity and **(B)** DNA fragmentation cell death ELISA. **(C)** Mino dose–response. Rats were IP injected twice daily with PBS, 2.5 mg/kg (Mino-5), 7.5 mg/kg (Mino-15) or 22.5 mg/kg (Mino-45) beginning 24 h previous to ischemia, and 48 h following IR retinas were assayed by DNA fragmentation cell death ELISA. Results shown are the means and standard error of means obtained from eight animals per group. *P ≤0.05, **P ≤0.01 and ***P ≤0.001 by Student’s *t*-test.

To test the effect of Mino treatment on retinal cell death at 48 h following IR the previously established [7] endpoints of caspase-3 activation and DNA fragmentation were employed. Mino failed to significantly affect these indicators of neurodegeneration (Figure 5A and B). Because of evidence that 5 to 10 times lower doses of Mino can provide greater neuroprotection (reviewed in [24]) we performed a dose–response experiment employing a dose essentially the same as prior experiments but lacking the increased loading dosage, as well as doses 3 times and 9 times lower than before. None of these doses of Mino significantly inhibited apoptosis (Figure 5C). Furthermore, while local intravitreal injection of Mino significantly (*P* <0.05) prevented IR-induced vascular permeability to a similar extent as systemic delivery of the drug, this treatment had no significant effect on DNA fragmentation or accumulative measures of neurodegeneration, including retinal layer thinning or the reduction of the ERG b-wave amplitudes measured at 2 wk and 1 wk following IR, respectively (see Additional file 3: Figure S1). Thus, Mino treatments that significantly reduced vascular permeability and inflammatory responses had no significant effect on neurodegeneration in this model.

## Discussion

Mino reduced IR-induced neuroinflammation, including the expression of inflammatory genes and leukostasis, and prevented IR-induced permeability and tight junction disorganization. To the best of our knowledge the present study is the first to examine the effects of Mino on vascular permeability and cellular inflammation following retinal IR. In fact, there are very few published studies of any kind on Mino’s effects on retinal vascular permeability. Chen and coworkers [37] demonstrated that Mino treatment diminished Evans blue dye leakage following repeated intravenous injection of glycated albumin into rats [37]. Using OCT, Sun *et al*. observed that Mino treatment significantly inhibited edema in the inner retina at 3 d following branch retinal vein occlusion (BRVO) [38]. On the other hand, several studies have demonstrated that Mino reduced brain edema in models of cerebral IR [39-41], stroke [42-44], as well as traumatic brain injury, infection and neurodegeneration [45-48]. Intriguingly, a small clinical study of five patients with diabetic macular edema found that 6 mo of Mino treatment reduced vascular fluorescein leakage and diminished mean retinal thicknesses [49].

The lack of neuroprotection by Mino observed in the present study is surprising because Mino is well known as a neuroprotective agent (reviewed in [50]). Mino was not significantly neuroprotective according to measures at 48 h following IR (caspase-3 activity and DNA fragmentation, Figure 5), at 24 h following IR (DNA fragmentation, (see Additional file 3: Figure S1B)) and by accumulative measures at 1 to 2 wk following IR (ERG and retinal layer thinning, (see Additional file 3: Figures S1C and D)). Previously, Mino inhibited retinal neurodegeneration in models of diabetic retinopathy [25], light induced retinopathy [26,27], glaucoma [28,29], axotomy [28,30] and retinal detachment [31]. Mino also inhibited the death of retinal neurons induced by glutamate and trophic factor withdrawal [51]. More applicable to the ischemic retina, Mino had small but significant effects on RGC loss and IPL thinning, while failing to prevent loss of ERG b-wave responses, in a rat model of BRVO [38]. In direct contrast to the present results, Mathalone and colleagues observed that systemic Mino treatment protected against RGC loss and inner retinal layer thinning following IR in the rat [52]. The reasons for this discrepancy are unknown. Matholone *et al*. employed relatively low Mino doses of 2.5 and 5 mg/kg-day and a 90 min ischemic insult that resulted in almost total disappearance of the IPL, rather than the 21% reduction in IPL thickness observed herein (see Additional file 3: Figure S1). While lower doses of Mino may actually be more neuroprotective than 45 mg/kg-day [24], in the present study Mino doses of 15 mg/kg-day and 5 mg/kg-day also failed to significantly inhibit DNA fragmentation following IR (Figure 1D). Thus, the dosing regimen is unlikely the reason for lack of neuroprotection. Regardless of the reason for the observed lack of neuroprotection, the present results demonstrate that inflammatory and vascular response can be disassociated from the neurodegenerative response to IR.

It is possible that Mino’s ability to inhibit inflammation and inflammatory cell attraction may reduce leakage at 24 to 48 h after IR by averting endothelial damage caused by adherent or invading leukocytes. However, the data does not provide direct evidence of a linkage between leukostasis and vascular dysfunction. Such evidence was presented by Hirata and co-workers [22] who used silver nitrate staining of endothelial cell boundaries and an antibody to CD45 to show that a Rho-associated kinase inhibitor, Y-27632, blocked leukostasis that was spatially associated with gaps in the endothelial layer following rat retinal IR. In contrast to Mino treatment, Y-27632 also inhibited ganglion cell loss and IPL thinning following IR. Although we did not observe apparent gaps in the vascular endothelium following IR, it is possible that damage to the endothelium contributes to leakiness following retinal IR. Danesh-Meyer and colleagues observed evidence of endothelial cell loss following retinal IR [8]. These authors found that inhibition of connexin43 gap junction activity prevented both neuronal cell death and vascular leakage. Similarly, Krueger and co-workers recently found that leakiness of the BBB in a stroke model was primarily located in regions with intact tight junctions and with evidence of increased transcellular vesicle trafficking and endothelial cell disintegration [53]. The failure of Mino to prevent leakage immediately after IR (see Additional file 4: Figure S2) suggests that early and late permeability are caused by different mechanisms. We recently found that vascular leakiness immediately following IR coincided with phosphorylation and ubiquination of the tight junction protein occludin (A. Muthusamy *et al*., in press). Thus, it is possible that Mino treatment inhibits vascular permeability by protecting tight junction alterations (as observed in this study), and also by inhibiting transcellular trafficking and preventing endothelial cell death (not directly investigated in this study). Future studies using biochemical and genetic approaches specifically targeting the vascular endothelium or infiltrating leukocytes may allow further testing of a causal relationship between inflammation and endothelial cell destruction and/or disruption of the BRB. Prevention or inhibition of inflammation may reduce permeability by inhibiting all of these potential routes of flux.

IR induced neuroinflammation that was inhibited by Mino treatment. We developed a set of 25 mRNA markers to monitor the inflammatory and astrogliosis responses of the retina to IR injury. The responses at 48 h following IR were very different from those observed after intravitreal injection of LPS, which represents a classical inflammatory insult mediated by activation of toll like receptor 4 (TLR4). The expressions of mRNAs corresponding to genes associated with classical inflammation (ICAM-1, CCL2, IL6, IL1B, TNF and CXCL2) were significantly increased in IR. However, the induction of these mRNAs by IR was dwarfed by the induction observed following intravitreal LPS injection. Several studies have documented induction of classical pro-inflammatory genes, including NOS2, COX2, TNF-α, IL-1β, and IL-6, in rodent retinas within hours of IR [11-17]. The lack of induction of NOS2, COX2, CCL5, and CXCL10 mRNAs 48 h following IR, as well as the relatively small induction of TNF-α, IL-1β, IL-6 and CXCL2 suggests a fundamental difference from classical inflammation. Several mRNAs indicative of neuroinflammation were significantly upregulated, including SERPINA3N, TNFRSF12A, endothelin 2 (EDN2), sphingokinase 1 (SPHK1), CHI3L1 and LGALS3. Intravitreal injection of LPS caused either much less induction or no induction of these neuroinflammatory markers. Thus, the response to IR may be more characteristic of the non-classical neuroinflammation that has been termed ‘pseudo-inflammation’ and has been largely attributed to the response of the innate immune system composed of glial and microglial cells [54]. The significant induction of GFAP and VIM mRNAs, as well as the significant reduction of GLUL mRNA, suggests that Müller cells, and possibly astrocytes undergo astrogliosis following IR.

Mino significantly inhibited the increase in expression of CCL2 and ICAM1 mRNAs. It is likely that the inhibition of expression of CCL2 and ICAM1 contributed to the inhibition of leukostasis by reducing the attraction and adhesion of leukocytes to the retinal vascular endothelium. Retinal IR injury upregulates both P-selectin and ICAM1 expression, presumably leading to increased leukocyte rolling and adhesion on the endothelial lumen [55]. Blocking antibodies to P-selectin or ICAM-1 also inhibited leukostasis and retinal layer thinning following IR [56]. We did not include P-selectin in our set of IR-responsive mRNA markers, as the original transcriptomic analysis did not identify it as being significantly altered by IR [7].

Flow cytometric quantification of CD11b and CD45 leukocyte markers was used to quantify the accumulation of immune cells in the retina following IR. Resident microglia constitute the vast majority of the CD11b^+^/CD45^low^ population in retina and the central nervous system [57-61]. We observed a slight but significant 30% increase in the number of CD11b^+^/CD45^low^ cells following IR, which was not affected by Mino treatment. We do not know if this apparent increase represents proliferation of the resident microglial population or an influx of circulating monocytes. There was a very significant increase in the number of CD11b^+^/CD45^hi^ cells following IR. CD11b positivity with high levels of CD45 is a classic indicator of myeloid leukocytes, which include mature macrophages, monocytes, granulocytes (primarily neutrophils) and dendritic cells. It is probable that retinal resident dendritic cells are included in this population. Indeed, in the basal state mouse retina contains approximately 100 dendritic cells per retina [62]. Using flow cytometry, we observed a highly significant accumulation of both CD11b^+^/CD45^hi^ myeloid leukocytes and CD11b^neg^/CD45^hi^ non-myeloid lymphocytes after IR. The accumulations of these populations were significantly attenuated by Mino treatment.

Expression of MHCII is a characteristic of antigen presenting cells (APC), including monocytes, macrophages, dendritic cells and B-lymphocytes. MHCII can also be expressed by activated T-cells [63]. Subdividing inflammatory cells into MHCII-positive and MHCII-negative groups revealed that Mino more significantly inhibited the accumulation of the MHCII^+^ subpopulations, suggesting that it acts to preferentially block the accumulation of inflammatory leukocytes. The seemingly preferential action of Mino may also be due to a shift in MHCII expression. Mino inhibited the upregulation of MHCII expression in microglia and macrophages during activation by gamma-interferon [64]. Thus, it is possible that Mino inhibited the accumulation of both MHCII positive and negative leukocytes equally, but also shifted MHCII^+^ populations toward a less activated state.

The molecular mechanism by which Mino may act to reduce inflammation and vascular permeability following IR is uncertain. In addition to its bacteriostatic capabilities, Mino is endowed with several functional properties that lead to pleiotropic effects [23]. Mino inhibits caspase-1 mRNA expression in cerebral IR [65] and a Huntington disease model [66]. Caspase-1 is also known as interleukin-1β converting enzyme (ICE), an integral part of the inflammasome. Mino inhibited caspase-1 activity and IL-1β expression in retinas of diabetic mice [67]. Mathalone and colleagues argued that Mino’s ability to inhibit metalloproteinase activities was responsible for the neuroprotective effects of Mino following IR in the rat [52]. Metalloproteinases have also been implicated in disruption of the BBB and permeability in cerebral ischemia (for review see [68]). Due to its phenolic rings and dimethylamino group on a phenolic carbon, Mino also acts as an effective scavenger of reactive oxygen species (ROS) [69]. Given that ROS are implicated in the mechanism of vascular dysfunction following IR [70], the ability of Mino to scavenge ROS could also conceivably account for its vascular protective abilities following IR.

## Conclusions

Retinal diseases invariably involve a combination of neurodegeneration, vascular dysfunction and inflammation in various proportions. The retinal IR injury model has primarily been used to explore means to prevent the neurodegenerative response triggered by a transient ischemic insult, but it also provides a useful means to explore the prevention of vascular and inflammatory responses to injury. Although Mino can undoubtedly be neuroprotective, the present study found that Mino diminished retinal neuroinflammation, leukostasis and vascular leakage without affecting indicators of astrogliosis or neuronal cell death. The molecular mechanisms by which Mino mediated the inflammatory and vascular responses to IR were not identified, but we can reasonably conclude that this was not the result of reducing the extent of neuronal damage caused by IR. The ability of Mino to inhibit the expression of chemokines such as CCL2 and adhesion molecules such as ICAM-1 would be expected to diminish leukostasis. Further studies are needed to determine the extent to which inflammatory gene expression and/or leukostasis following IR causes vascular dysfunction and leakage, perhaps by damaging the endothelium. Our data suggest that there is also a direct effect of IR on endothelial tight junction organization. A remaining issue is the extent to which leakiness is caused by inflammation, perhaps by leukocyte-induced collateral damage to the endothelium, versus the extent of leakiness caused by inflammation-independent effects on endothelial cells. The contributions of these mechanisms may indeed change over time.

## Abbreviations

BBB: Blood–brain barrier; BRB: Blood-retinal barrier; CNS: Central nervous system; ERG: Electroretinogram; GCL: Ganglion cell layer; IF: Immunofluorescence; INL: Inner nuclear layer; IOP: Intraocular pressure; IP: Intraperitoneal; IPL: Inner plexiform layer; IR: Retinal ischemia; LPS: Lipopolysaccharide; Mino: Minocycline; NT: Nontreated animals; OCT: Optical coherence tomography; ROS: Reactive oxygen species; RT: Room temperature.

## Competing interests

The authors have no competing interests to declare.

## Authors’ contributions

SFA designed experiments, produced figures and wrote the manuscript. CML designed experiments, performed experiments, produced figures and helped write the manuscript. SS performed experiments and produced figures, AM designed experiments, performed experiments, produced figures and edited the manuscript. AJB designed experiments, performed experiments, produced figures and edited the manuscript. DAA designed experiments and co-wrote the manuscript. All authors read and approved the final manuscript.

## Supplementary Material

Additional file 1 Supplemental Methods. Click here for file

Additional file 2: Table S1 Effect of minocycline (Mino) treatment on neuroinflammatory gene and astrogliosis-related gene expression. Mino was delivered as twice-daily intraperitoneal (ip) injections, with two initial dosages of 45 mg/kg prior to ischemia and dosages of 22.5 mg/kg just prior to ischemia and every 12 h for the next 2 d during the reperfusion period as described in Materials and Methods. One eye of each rat was subjected to retinal ischemia (IR) for 45 min or needle puncture only (Sham) and after 48 h of reperfusion total RNA was isolated from retinas and relative mRNA levels of 25 genes shown was assayed by duplex qRT-PCR with β-actin mRNA serving as an internal control. Gene symbols, Applied Biosystems (ABI) assay numbers and calculated efficiency of primers obtained in duplex assays with β-actin primer/probes (or for β-actin primer alone) are shown. Mean mRNA levels are all relative to that of the no-treatment (NT) Sham group. Results for IR experiments are the means and standard error of means obtained from eight animals per group. Percent inhibitions by Mino are calculated from effects of IR observed in Mino-treated and non-treated rats ((1 – (Mino-IR – Mino-Sham)/(NT-IR – NT-Sham)) × 100%). For comparison, × the mean induction of expression mRNAs in retinas (n = 3) at 4 h following intravitreal injection of 1 μg/eye of lipopolysaccharide (LPS) is shown. *P* values for statistical comparisons of means were calculated using Students *t*-test. Click here for file

Additional file 3: Figure S1 Intravitreal injection of minocycline (Mino) inhibited retinal vascular leakage but did not prevent layer thinning or electroretinogram (ERG) deficits following retinal ischemia (IR). Each animal in the treatment group was injected with 640 ng of Mino at 4 h prior to IR and 1 h after reperfusion. Non-treated rats were injected with PBS. One eye of each animal was subjected to retinal ischemia for 45 min and reperfused for 24 h. The contralateral eye was subjected to needle puncture only and served as sham control. A) Twenty-four hours after reperfusion retinas were assayed for Evans blue dye leakage as described in Materials and Methods. B) Twenty-four hours after reperfusion retinas were assayed for as DNA fragmentation described in Materials and Methods. C) Two weeks after reperfusion, retinal inner plexiform layer (IPL) thicknesses were measured from cross sections and the ratios of IPL/(OPL + ONL) calculated to represent IPL thicknesses corrected for non-perpendicular sectioning angles. Results shown are the means and standard error of means obtained from eight animals per group. *P ≤0.05, **P ≤0.01 and ***P ≤0.001 by Students *t*-test. D) One week after ischemia ERGs were recorded. The b-wave amplitudes for increasing flash intensities are shown. Using the mixed effects two-way analysis of variance (ANOVA) model to compare best fits of the data, the effect of IR was significant (*P* <0.05) for both Mino and PBS treated eyes, where there was no significant difference between IR groups of Mino and PBS treated eyes. Click here for file

Additional file 4: Figure S2 Mino treatment did not inhibit retinal vascular leakage immediately following retinal ischemia (IR). Mino was delivered as twice-daily intraperitoneal (ip) injections, with two initial dosages of 45 mg/kg prior to ischemia and a dosage of 22.5 mg/kg just prior to ischemia as described in Materials and Methods. Non-treated (NT) animals received PBS vehicle injections. One eye of each rat was subjected to retinal ischemia (IR) for 45 min or needle puncture only (Sham) and after 15min of reperfusion retinas were assayed for Evans blue dye leakage (n = 7 to 8 retinas per group). Evans blue dye was injected 15 min after IR and circulated for 2 h prior to flushing and removal of retinas. ns = not significant, **P ≤0.01 and ***P ≤0.001 by Student’s *t*-test. Click here for file
