# Supplementary material for: Minocycline prevents retinal inflammation and vascular permeability following ischemia-reperfusion injury
Source: J Neuroinflammation. 2013 Dec 10;10:149. doi: 10.1186/1742-2094-10-149 (PMC3866619; doi:10.1186/1742-2094-10-149)
Supplement: Additional file 1 — Supplemental Methods. [file 1742-2094-10-149-S1.docx]

SUPPLEMENTAL METHODS:

*Intravitreal injection of minocycline (Mino):* 640 ng of Mino dissolved in 2µl of PBS and at a neutral pH was injected using a 32-gauge needle 4 h prior to ischemia and 1 h after reperfusion. PBS alone (2 µl/eye) was injected in treatment control eyes.

*Analysis of electroretinagram (ERG):* ERG recordings were made using an Espion2 with Colordome stimulator and 37°C heating platform (DiagnoSYS, www.diagnosysuk.com). Rats were dark adapted overnight and then all procedures were performed under red light (628 nm LED bulb). Rats were anesthetized with ketamine and xylazine (66.7 mg/kg and 6.7 mg/kg body weight, respectively). Pupils were dilated with 1% tropicamide/1% atropine (Bausch and Lomb, Tamps, FL) and both corneas were coated with GenTeal gel (Novartis, www.novartis.com). A monopolar corneal contact lens electrode was used for recording (LKC Technologies, www.lkc.com), with flat crocodile clips on the cheek and hind limb as reference and ground electrodes, respectively. The ERG response to a sequence of scotopic flashes of white light (0.01 to 100 cd•s/m^2^) was recorded, beginning with the lowest energy stimulus. At the lowest stimulus, 10 sweeps were averaged. The next three stimuli were averaged 5 times and single responses were recorded for the two highest stimuli. The interstimulus interval ranged between 2 sec and 3 min according to the stimulus intensity. Responses were digitally filtered from 0.625 to 1,000 Hz and amplified 1,000 times. B-wave amplitudes began to increase with increasing light stimulus above 0.1 cd•s/m^2^; therefore the implicit time and amplitude of the a- and b-waves were calculated for stimulus intensities of 0.1 to 100 cd•s/m^2^. For statistical analysis of treatment effects on ERG b-wave intensities, a linear mixed model was fit to the mean b-wave amplitudes of each group as a function of light stimulus intensity. The linear trend was appropriate based on mean response plots and residual analysis. An unstructured covariance was used for the first source of correlation (between sham and ischemic eyes within an animal) and an autoregressive structure with a lag of 1 was used for the second source of correlation (within an animal over stimulus intensity). The p-values were calculated from the final correlation matrix estimated by the model, which was the direct product between the unstructured and autoregressive covariance components.

*Retinal layer thinning:* Whole eyes were fixed by immersion in 4% paraformaldehyde (20 min, room temp.) and then flash frozen. Four sagittal paraffin sections (10 µm) were made through the center of the eye, which included the optic nerve. Sections were stained with hematoxylin and eosin (H&E) and examined on an Olympus BH-2 microscope (www.olympusamerica.com) fitted with Sony CCD video camera. The thickness of the inner plexiform layer (IPL), inner nuclear layer (INL) and combined outer plexiform layer and outer nuclear layer (OPL+ONL) was measured in regions approximately 200 µm on either side of the optic nerve, using Optimus (Media Cybernetics, www.mediacy.com) calibrated to a micrometer. The averaged data were expressed as a ratio of IPL /(OPL+ONL) to avoid variation due to non-perpendicular sectioning angle, and because the largest decrease in thickness was across the IPL, whereas the combined thickness of OPL+ONL was not significantly altered by IR. Statistical differences between like-treated Sham and IR retina groups were analyzed by paired Student’s *t*-test, with effects of treatments analyzed by unpaired Student’s *t*-test.
