# Supplementary figures and images for: Minocycline prevents retinal inflammation and vascular permeability following ischemia-reperfusion injury
Source: J Neuroinflammation. 2013 Dec 10;10:149. doi: 10.1186/1742-2094-10-149 (PMC3866619; doi:10.1186/1742-2094-10-149)

A

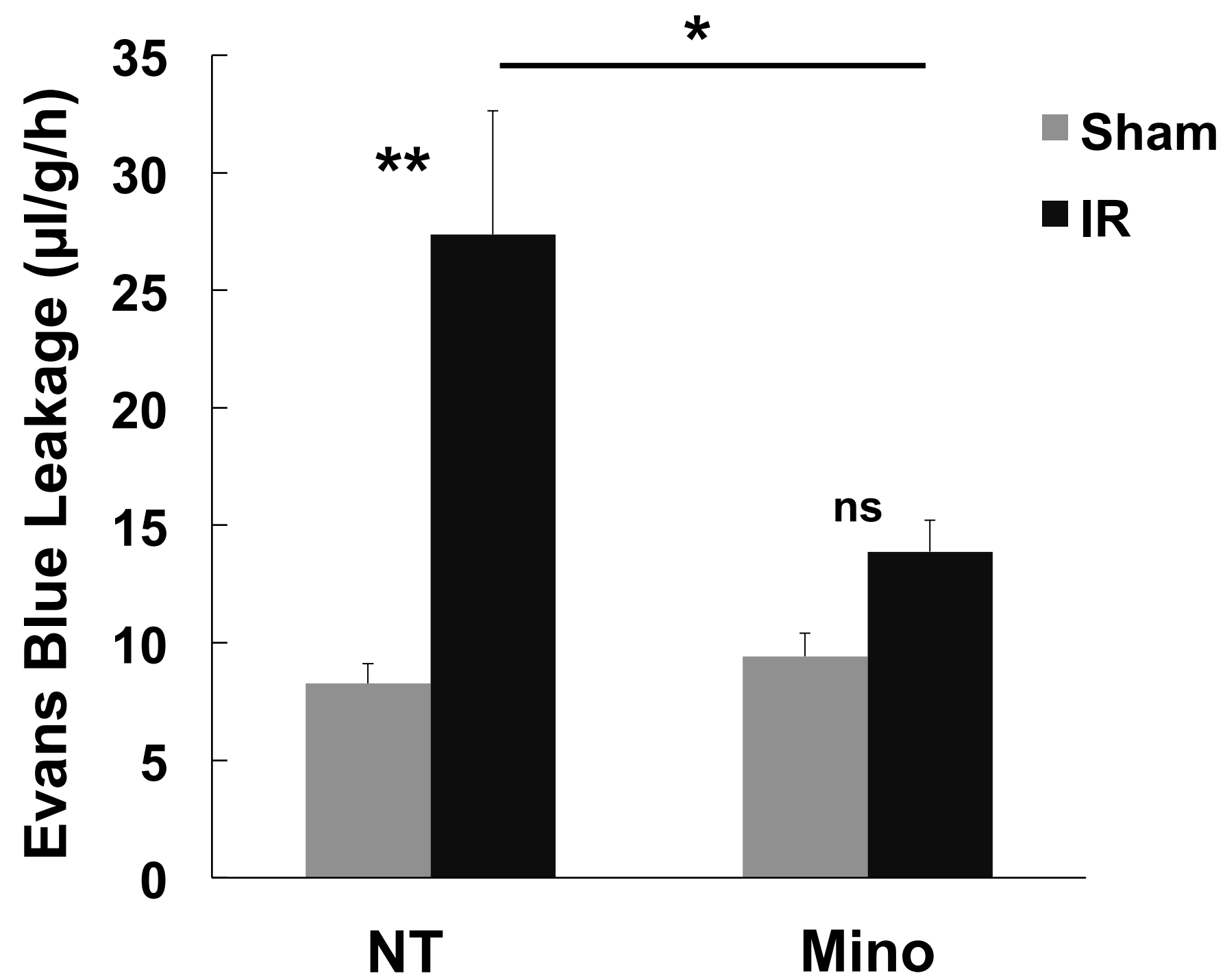

B

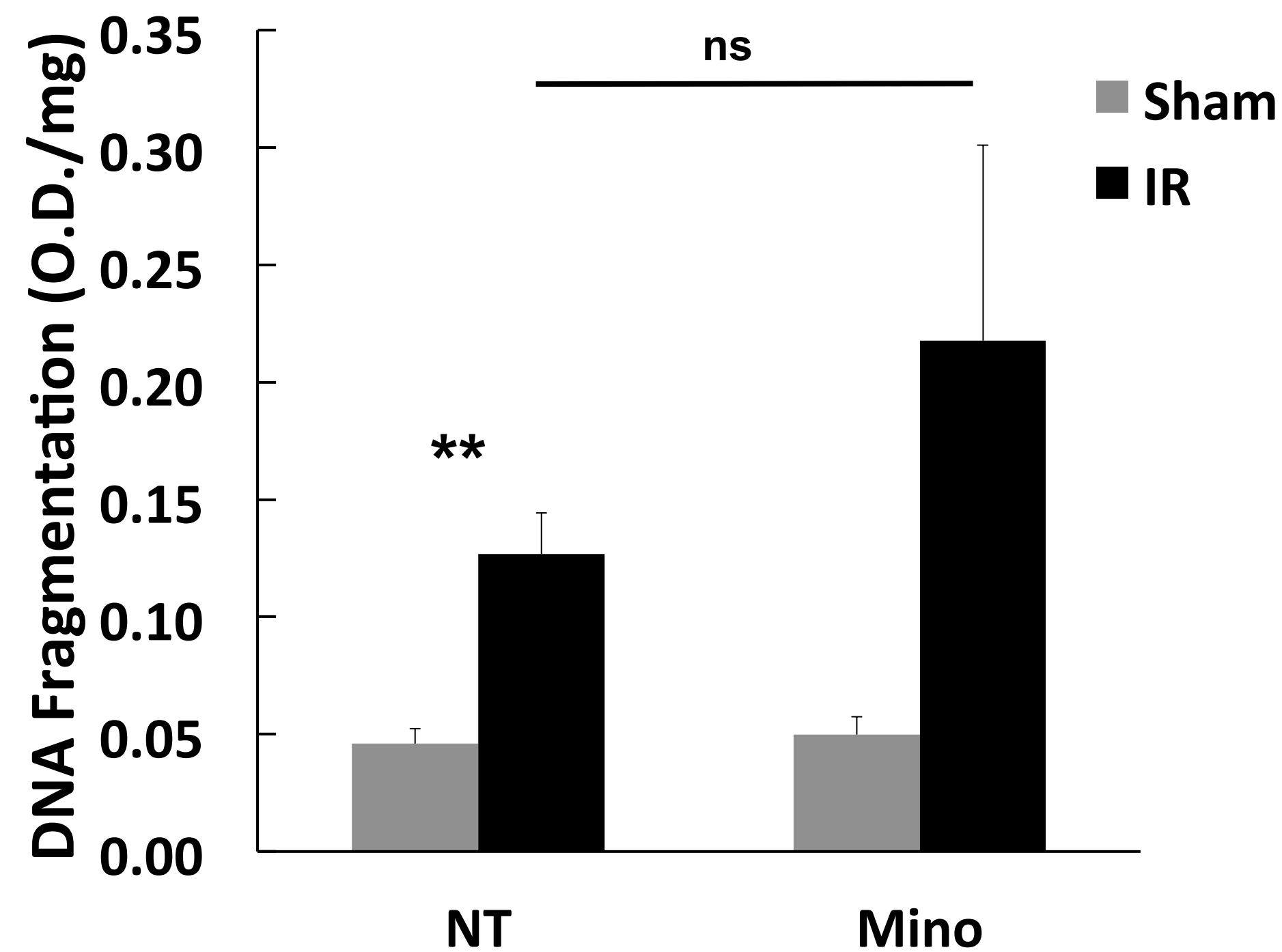

C

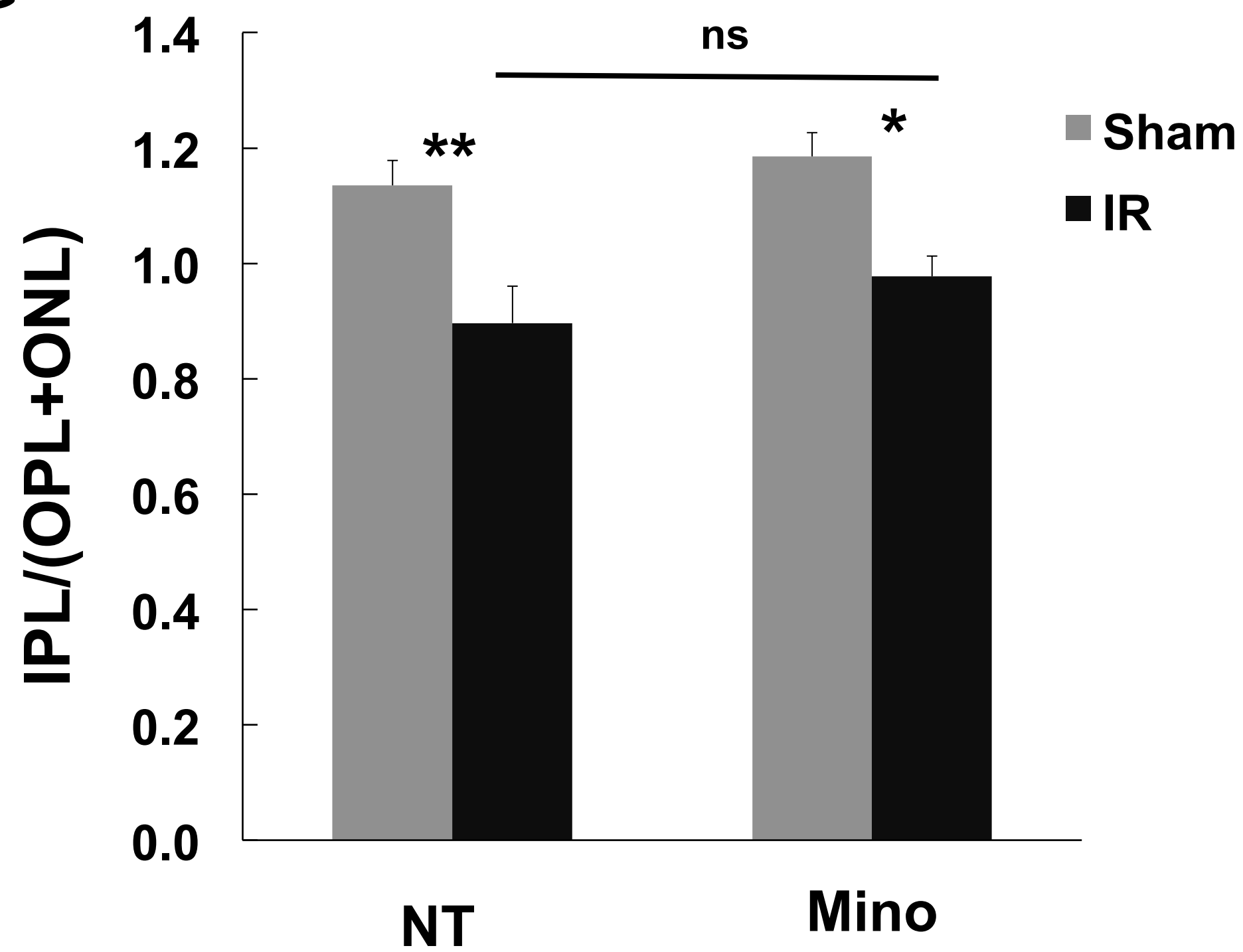

D

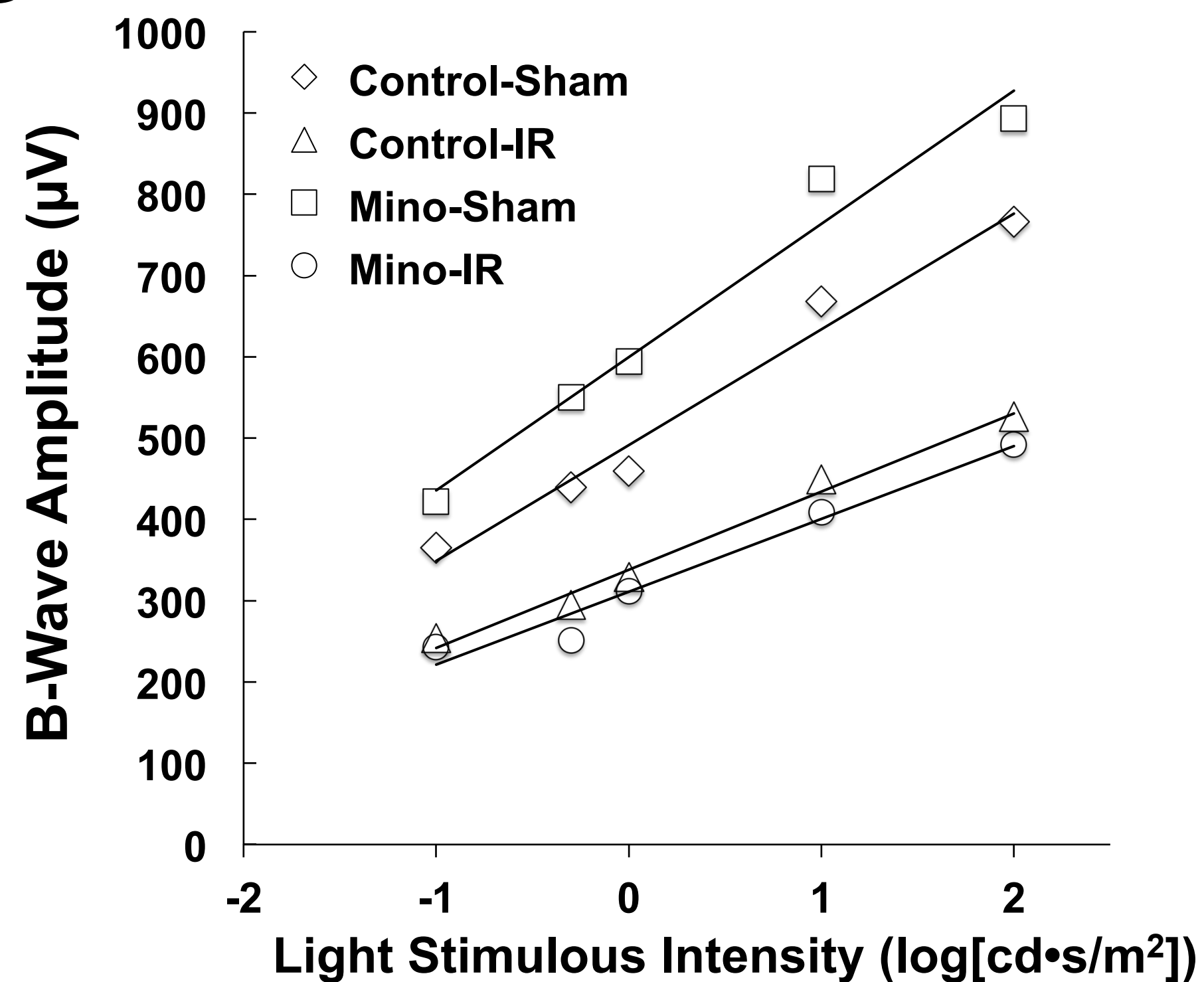

Supplement: Additional file 3: Figure S1 — Intravitreal injection of minocycline (Mino) inhibited retinal vascular leakage but did not prevent layer thinning or electroretinogram (ERG) deficits following retinal ischemia (IR). Each animal in the treatment group was injected with 640 ng of Mino at 4 h prior to IR and 1 h after reperfusion. Non-treated rats were injected with PBS. One eye of each animal was subjected to retinal ischemia for 45 min and reperfused for 24 h. The contralateral eye was subjected to needle puncture only and served as sham control. A) Twenty-four hours after reperfusion retinas were assayed for Evans blue dye leakage as described in Materials and Methods. B) Twenty-four hours after reperfusion retinas were assayed for as DNA fragmentation described in Materials and Methods. C) Two weeks after reperfusion, retinal inner plexiform layer (IPL) thicknesses were measured from cross sections and the ratios of IPL/(OPL + ONL) calculated to represent IPL thicknesses corrected for non-perpendicular sectioning angles. Results shown are the means and standard error of means obtained from eight animals per group. *P ≤0.05, **P ≤0.01 and ***P ≤0.001 by Students t-test. D) One week after ischemia ERGs were recorded. The b-wave amplitudes for increasing flash intensities are shown. Using the mixed effects two-way analysis of variance (ANOVA) model to compare best fits of the data, the effect of IR was significant (P <0.05) for both Mino and PBS treated eyes, where there was no significant difference between IR groups of Mino and PBS treated eyes. [file 1742-2094-10-149-S3.pdf]

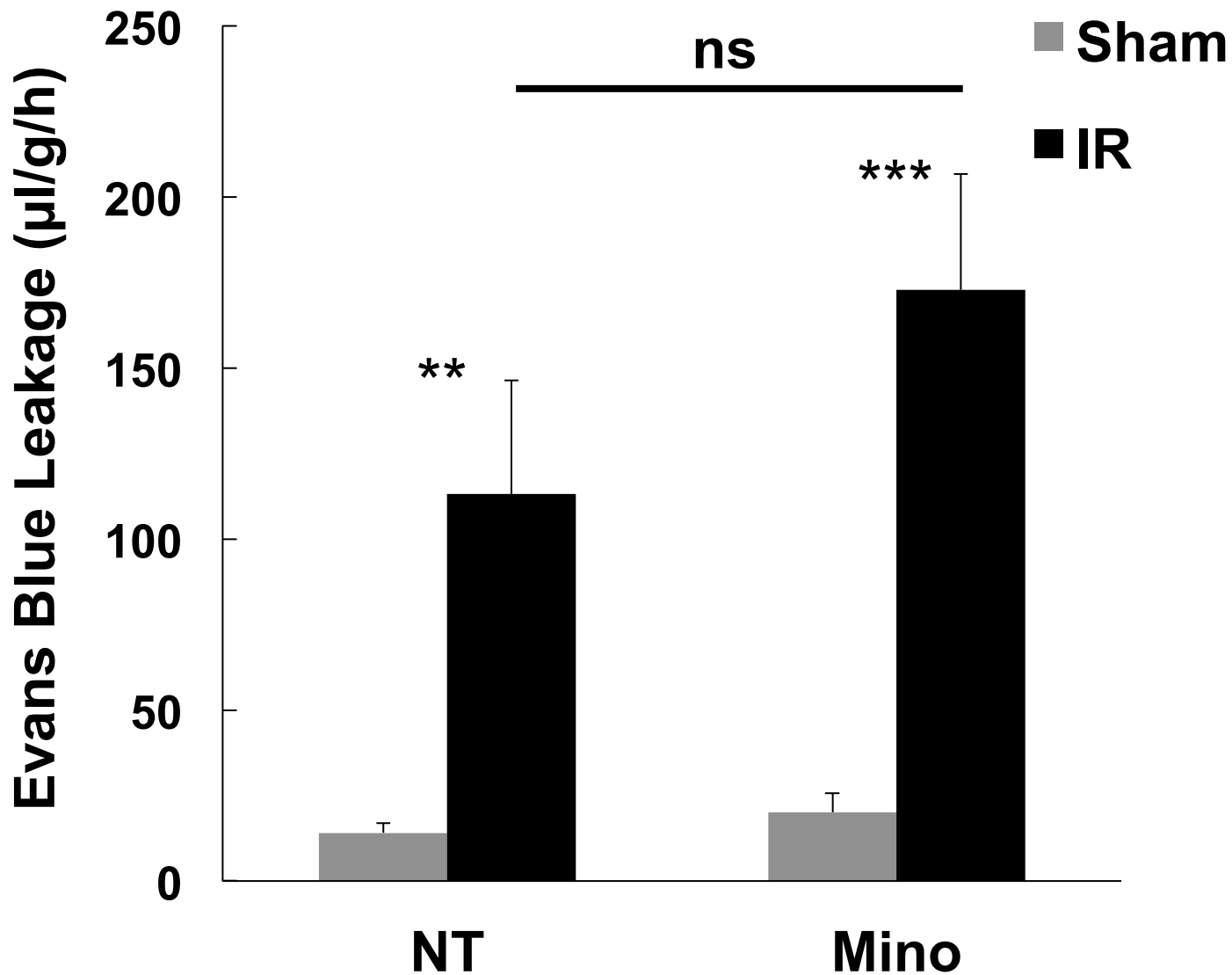

Supplement: Additional file 4: Figure S2 — Mino treatment did not inhibit retinal vascular leakage immediately following retinal ischemia (IR). Mino was delivered as twice-daily intraperitoneal (ip) injections, with two initial dosages of 45 mg/kg prior to ischemia and a dosage of 22.5 mg/kg just prior to ischemia as described in Materials and Methods. Non-treated (NT) animals received PBS vehicle injections. One eye of each rat was subjected to retinal ischemia (IR) for 45 min or needle puncture only (Sham) and after 15min of reperfusion retinas were assayed for Evans blue dye leakage (n = 7 to 8 retinas per group). Evans blue dye was injected 15 min after IR and circulated for 2 h prior to flushing and removal of retinas. ns = not significant, **P ≤0.01 and ***P ≤0.001 by Student’s t-test. [file 1742-2094-10-149-S4.pdf]
